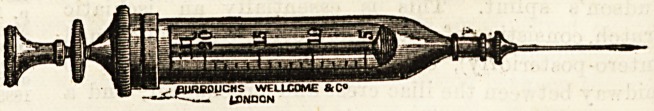# New Appliances and Things Medical

**Published:** 1894-02-17

**Authors:** 


### NEW APPLIANCES AND THINGS MEDICAL.

### SILK'S CELLULOID ETHER INHALER.

(Down Brothers, 5 and 7, St. Thomas Street, Borough.)

Following the instructions of Dr. Silk, the above manufacturers have taken a new departure in the matter of mouth pieces for the administration of anæsthetics. It has long been a subject of annoyance to anæsthetists that the india-rubber or leather of which the mouthpieces are usually composed, soon becomes tainted with a most disagreeable odour, or fall to pieces in the attempt to keep them sweet by frequent washings. The new celluloid mouthpiece offers the following advantages: They can be kept perfectly inodorous by washing in cold water, they are light, cleanly, and inexpensive. The makers undertake to manufacture celluloid mouthpieces to fit any inhaler at the same price as the usual leather mouthpiece, but for ordinary purposes the celluloid inhaler for ether and A. C. E., price 10s. 6d., is the cheapest and cleanest form of inhaler with which we are acquainted.

### "THE PURE ALUMINIUM" HYPODERMIC SYRINGE.

(Burroughs, Wellcome, and Co., Snow Hill Buildings, London, E.C.)

Messrs. Burroughs and Welcome have forwarded us a specimen of their new patent aseptic hypodermic syringe for inspection and trial; we have made a careful examination of the same, and have subjected it to a few searching tests. We are able to record a very favourable impression; it certainly stands a satisfactory comparison to other syringes designed with the same objects in view. Its chief merits are simplicity of structure, and the facility with which it may be employed. There is no excuse for a nurse or even a student going wrong in the manipulation or cleansing of this little instrument after reading the instructions. The syringe, after or before use, should be taken to pieces, boiled in water, and subsequently rinsed with absolute alcohol; it is thus rendered perfectly aseptic. The washer or packing of the piston can readily be adjusted and made to fit the barrel by manipulating a small nut situated directly beneath the knob of the piston rod. Blood poisoning from the use of contaminated hypodermic syringes is fortunately a rare event, but accidents of this nature have occurred in the past. If, however, they occur in the future, with so ready a prophylactic means as that afforded by this new syringe, there can be no possible excuse for such carelessness. In America a practitioner so doing might be successfully sued in a court of law for heavy damages. The makers claim for their invention a unique degree of lightness owing to the nature of the aluminium case which ensheathes the barrel. The syringe with two needles costs only 7s. Extra barrels to fit the case may be procured for 1s. each, and the washers or packings for the piston rod cost 2s. a dozen. The syringe in our possession is fitted in a neat little case ("The Miniature"), and together with partitions for 15 tubes of hypodermic tabloids is sufficiently small to be carried with convenience in the waistcoat pocket.

## Figures and Tables

**Figure f1:**
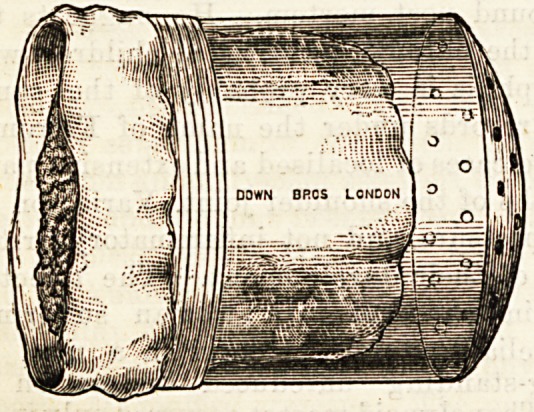


**Figure f2:**
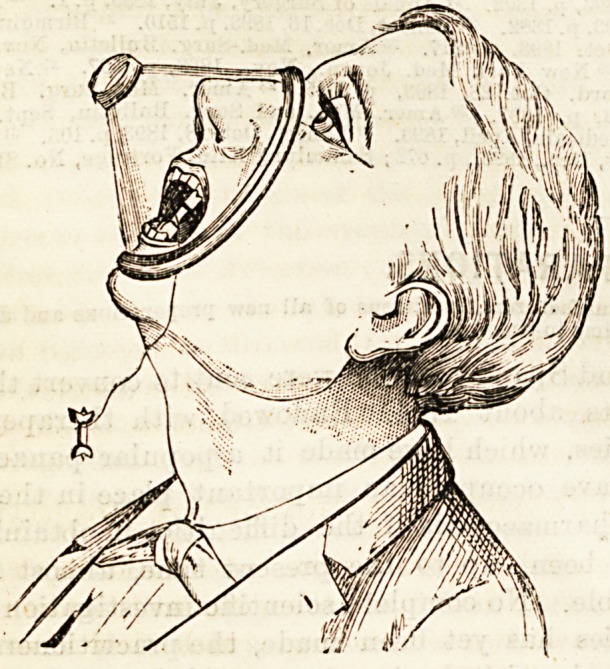


**Figure f3:**